# CT-guided vs. fluoroscopically guided transforaminal epidural steroid injections for lumbar radiculopathy: a comparison of efficacy, safety and cost

**DOI:** 10.1007/s00402-022-04436-y

**Published:** 2022-04-14

**Authors:** Jozef P. M. Kamp, Jonathan Bartlett, Amr Fahmy, Kendrick To, Rumana Hossain, Maheswara Akula

**Affiliations:** 1 Department of Trauma and Orthopaedics, Basildon and Thurrock University Hospital, Basildon, UK; 2 Department of Trauma and Orthopaedics, Rotorua Hospital, Rotorua, New Zealand; 3 Division of Trauma and Orthopaedics, Department of Surgery, University of Cambridge, Addenbrooke’s Hospital, Cambridge, UK

**Keywords:** Lumbar radiculopathy, CT-guided, Fluoroscopically guided, Epidural steroid injection, Cost-effectiveness

## Abstract

**Introduction:**

There are no formal guidelines for whether CT-guided or fluoroscopy-guided TFESI should be undertaken for patients with symptoms of lumbar nerve root irritation and corresponding nerve impingement. Here, we sought to compare the efficacy, safety and cost of computer tomography (CT)-guided and fluoroscopically guided transforaminal epidural steroid injection (TFESI).

**Materials and methods:**

All patients who underwent lumbar TFESI at our institution between June 2016 and June 2018 were identified. Six-week follow-up outcomes were categorised. The radiation doses and associated cost was retrieved from our institution’s costing system.

**Results:**

One hundred and sixteen patients were included (CT—50; fluoroscopy—56). There were no complications. More patients were discharged 6 weeks after CT-guided lumbar TFESI when compared with fluoroscopically guided TFESI (CT—23, fluoroscopy—14 (*P* = 0.027)). There was no difference in the number of patients who were referred to surgery (*P* = 0.18), for further pain management (*P* = 0.45), or for further TFESI (*P* = 0.43). The effective radiation dose was significantly higher for CT-guided TFESI (CT—5.73 mSv (3.87 to 7.76); fluoroscopy—0.55 mSv (0.11 to 1.4) (*P* < 0.01)). The total cost for CT-guided lumbar TFESI was £237.50 (£235 to £337), over £800 less than under fluoroscopic guidance (£1052 (£892.80 to £1298.00), *P* < 0.01)). Removing cost associated with staff and theatre use (staffing, theatre, medical indemnity and overheads) revealed CT-guided lumbar TFESI to be less expensive than if the procedure was fluoroscopy-guided—CT-guided: £132.6 (130.8 to 197.5); fluoroscopy: £237.4 (£209.2 to £271.9) (*P* = 0.019).

**Conclusions:**

CT-guided TFESI was associated with a higher discharge rate, a lower cost, but a ten times higher radiation dose when compared with fluoroscopically guided TFESI. Prospective studies are required to compare the efficacy of these procedures and to investigate how the radiation dose of CT-guided TFESI can be reduced without jeopardising efficacy or safety.

## Introduction

Transforaminal epidural steroid injection (TFESI) of the lumbar spine is an increasingly common procedure performed for patients with radicular pain secondary to nerve compression [1–5]. This procedure can be performed using either computerised tomography (CT) or fluoroscopic guidance in a reliable, quick and safe manner [3, 4, 6]. Although both these procedures have been shown to provide symptomatic benefit for the patient, there has been limited comparison between these two procedures in clinical practice. CT guidance has been increasingly adopted for this procedure in recent years owing to its greater imaging definition [5, 6]. This has, however, raised concerns regarding excessive radiation exposure to both the patients and the operator due to radiation exposure stochastic effects, including genetic mutation and carcinogenesis [3, 4].

As such, the relative efficacy, safety and cost of these two procedures remains unclear and there are no formal guidelines or clinical care pathways for patients with symptoms of lumbar nerve root irritation and corresponding nerve impingement on magnetic resonance imaging. The aim of this study was to compare the clinical outcomes, radiation dose and cost of CT-guided and fluoroscopically guided TFESI for patients with unilateral single-level lumbar spine radiculopathy at our institution.

## Methods and materials

### Participants

All patients who underwent CT-guided and fluoroscopically lumbar TFESI between June 2016 and June 2018 at our institution were identified. This interval was chosen due to the availability of electronic records and sufficient follow-up data. Patients who underwent non-lumbar, bilateral or multilevel TFESI were excluded. Patient who had undergone previous TFESI at any level were excluded. All patients undergoing lumbar TFESI had documented symptoms of lumbar nerve root irritation with corresponding nerve root impingement on magnetic resonance imaging. All patients were reviewed by one of two experienced orthopaedic spinal consultants who then booked patients for TFESI on the basis of history, examination and investigations. Patient symptoms must have been present for a minimum of 3 months and have been resistant to conservative measures including physiotherapy and analgesia. Patient assignment to either fluoroscopy-guided or CT-guided lumbar spine injection was dependent on availability of each procedure at the time of referral: patients were booked for the procedure with the shortest duration to the next available slot.

### CT-guided and fluoroscopy-guided procedures

CT-guided lumbar TFESI were performed by a single experienced radiologist using a General Electric Lightspeed VCT computerised tomography scanner (2010, United Kingdom). Fluoroscopically guided lumbar TFESI were performed in surgical theatre by one of two experienced spine consultants using a Phillips BV Endura C-arm fluoroscopy machine (2013, United Kingdom). All injections for both procedures were performed using a local anaesthetic (levobupivacaine), and a long-acting corticosteroid (2 mL of 40 mg/mL Kenalog). All patients were then followed-up at 6 weeks post-procedure consultations.

### Clinical outcomes

Patients’ electronic records were reviewed by two authors (JK and JB). Age, gender and BMI were recorded for all patients to assess any discrepancy between the two intervention groups. Records were reviewed for the presence of intra-procedural or post-procedural complications. Patient clinical outcomes at 6-week follow-up were categorised as follows: successfully discharged; booked for further lumbar TFESI; referred for surgery; referred for pain management; or scheduled for further follow-up.

### Radiation doses

Radiation doses received by the patient were retrieved from automatically generated imaging study reports in dose length product in mGy.cm or dose area product in mGy.cm^2^ for CT-guided and fluoroscopically guided procedures, respectively. To enable comparison between the two cohorts, these values were converted to the effective dose in millisieverts (mSv) using previously defined conversion factors [7].

### Cost data

Cost data for all patients were retrieved for each care episode from our institution’s patient level information costing system (IQVIA, Durham, North Carolina, USA). This system categorised and recorded staffing, imaging, theatre, ward (including recovery), pharmacy (including drugs), equipment, medical indemnity and overhead costs for each hospital admission for each patient. Duration of procedures was factored into costings within the categories above.

### Statistical analysis

All data were collected manually using Microsoft Excel (Microsoft, Washington, USA) and all statistical analyses performed using FigurePad™ (Prism 7; Graphpad Software Inc., La Jolla, CA, USA). Due to the non-parametric nature of the data collected, all data are presented as median (interquartile range). For patients’ age and BMI, the effective radiation dose received, and the cost per procedure, a Mann–Whitney test was used to compare the two groups. A 5 × 2 contingency table was produced for the number of procedures performed within each group for each vertebral level and a Chi Squared test used to assess for any difference between the two groups. For patients’ sex and each clinical outcome, 2 × 2 contingency tables were produced and Fisher’s Exact test was used to assess statistical significance. When comparing cost data between the two groups, a Kruskal–Wallis test with multiple comparisons was performed.

## Results

A total of 232 patients were identified as having undergone TFESI, 102 under CT guidance and 130 under fluoroscopic guidance in our defined period. 116 patients who underwent non-lumbar, bilateral or multilevel TFESI were excluded (CT—44 patients; fluoroscopy—72 patients), yielding a final dataset of 116 patients (CT—50; fluoroscopy—56). We found no statistical significance between the demographics of each group or the percentage of patients undergoing TFESI at each vertebral level (Tables 1 and 2).

**Table 1:** Patient demographics for patients who underwent CT-guided^a^ and fluoroscopically guided TFESI^b^

	CT	Fluoroscopy
Age (years)	53 (43–65)	47 (36–66)
Body mass index (kg/m^2^)	28.7 (26.7–32.0)	29.0 (27.0–31.0)
Male to female ratio (M:F)	25:31	20:30

^a^Computer tomography

^b^Transforaminal steroid injection

**Table 2:** Number of procedures performed for each vertebral level for CT-guided^a^ and fluoroscopically guided TFESI^b^

Vertebral level	CT	Fluoroscopy
L1/2	3	0
L2/3	3	1
L3/4	7	6
L4/5	25	26
L5/S1	12	23

^a^Computer tomography

^b^Transforaminal steroid injection

### Clinical outcomes

There were no intra-operative or post-operative complications documented for any patient in either group. We found significantly more patients were discharged at 6-week follow-up and significantly fewer patients were scheduled for further follow-up alone if they underwent CT-guided lumbar TFESI as compared with fluoroscopically-guided TFESI (discharged: CT—23, fluoroscopy—14 (*P* = 0.03); follow-up: CT—3, fluoroscopy—3 (*P* = 0.01)) (Table 3)).

**Table 3:** Clinical outcomes at 6-week follow-up consultations for CT-guided^a^ and fluoroscopically guided TFESI^b^

Outcome	CT (*n* = 50)	Fluoroscopy (*n* = 56)	*P*
Discharged	23	14	0.03
Further injection	11	6	0.18
Referred to surgery	7	12	0.45
Referred for pain management	6	10	0.43
Scheduled for follow-up	3	14	0.01

^a^Computer tomography

^b^Transforaminal steroid injection

We found no significant difference in the number of patients who were referred to surgery (CT—7, fluoroscopy—12), referred for further pain management (CT—6, fluoroscopy—10) or who underwent further TFESI (CT—11, fluoroscopy—6) between the two groups (surgery: *P* = 0.45, pain team: *P* = 0.43, further injection: *P* = 0.18).

### Radiation dose

**Fig. 1:**
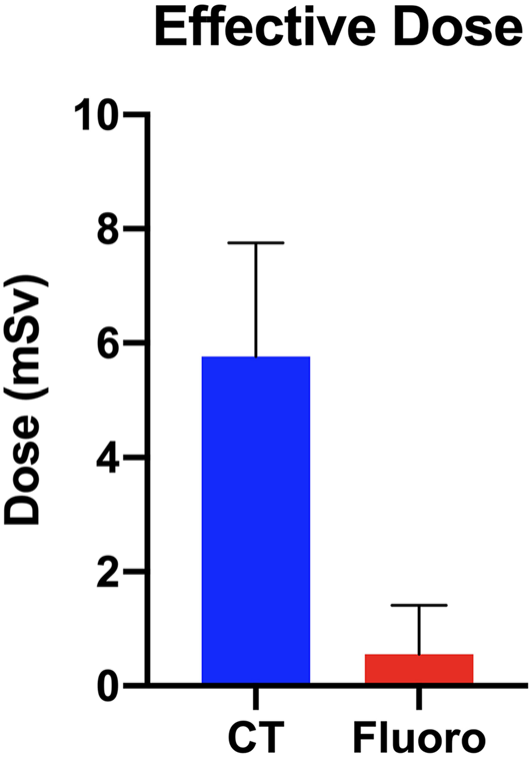
The median effective radiation dose received per patient for CT-guided versus fluoroscopically guided lumbar TFESI

The median effective radiation dose for patients who underwent CT-guided TFESI was 5.73 mSv (3.87 to 7.76). This was significantly more than the effective dose received by patients who underwent fluoroscopically guided TFESI which was 0.55 mSv (0.11 to 1.4, *P* < 0.01)) (Fig. 1).

### Cost of procedure

**Fig. 2:**
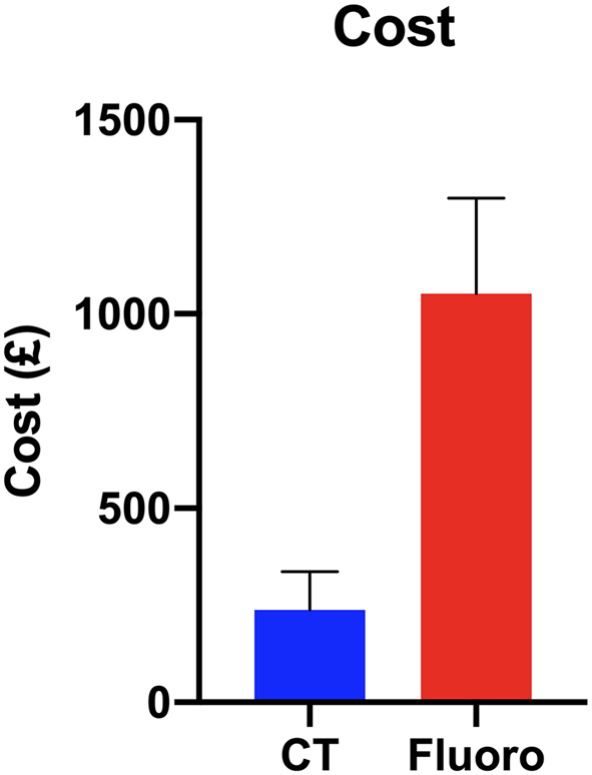
The median procedural cost per patient for CT-guided versus fluoroscopically guided lumbar TFESI

The median total cost for patients undergoing CT-guided lumbar TFESI at our institution was £237.50 (£234.70 to £337.30), over £800 less than for patients who underwent this procedure using fluoroscopic guidance (£1052.00 (£892.80 to £1298.00), *P* < 0.01)) (Fig. 2). Over 60% of this difference (£519.64) was attributed to the cost associated with staffing (£312.84) and operating theatre use (£206.80) (Table 4). Fluoroscopically guided TFESI was found to be more expensive than CT-guided TFESI in all costing categories (*P* < 0.01) excluding the cost of wards and recovery and the cost of imaging—in which no significant difference was found (Wards and recovery: *P* = 0.136; Imaging: *P* = 0.99)—and the cost of drugs and pharmacy services—in which CT-guided TFESI was found to be more expensive (*P* = 0.0396).

**Table 4:** Cost analysis for CT-guided^a^ and fluoroscopically guided TFESI^b^

	Median cost for CT-guided TFESI	Median cost for fluoroscopically guided TFESI	Difference in cost	*P*
Total	£237.50 (£234.70–£337.30)	£1,052.00 (£892.80–£1298.00)	£814.50	< 0.01
Clinical staffing	£33.66 (£33.66–£33.66)	£346.50 (£284.20–466.00)	£312.84	< 0.01
Imaging	£73.5 (£73.59–£73.59)	£71.17 (£71.17–£73.32)	£2.42*	0.99
Theatre	£0.00 (£0.00–£0.00)	£206.80 (£175.60–£239.60)	£206.80	< 0.01
Ward and recovery	£2.53 (£2.53–£61.93)	£2.53 (£2.53–£2.53)	£0.00	0.136
Pharmacy Cost	£27.83 (£27.83–£28.16)	£16.28 (£16.28–£17.44)	£11.55*	0.0396
Equipment	£26.84 (£25.03–£26.84)	£147.00 (£122.10–£171.10)	£120.16	< 0.01
Overheads	£55.73 (£54.90–£66.69)	£200.70 (180.10–£240.0)	£144.97	< 0.01
Medical indemnity	£15.95 (£15.95–£15.95)	£74.36 (£52.58–£101.10)	£58.41	< 0.01

*CT-guided TFESI was more costly than fluoroscopically guided TFESI

^a^Computer tomography

^b^Transforaminal steroid injection

Removing cost associated with staff and theatre use (staffing, theatre, medical indemnity and overheads) revealed CT-guided lumbar TFESI to be significantly less expensive than if the procedure was fluoroscopy guided—CT-guided: £132.6 (130.8 to 197.5); fluoroscopy: £237.4 (£209.2 to £271.9) (*P* = 0.019).

## Discussion

At our institution, we found significantly more patients were discharged at 6 weeks after CT-guided TFESI of the lumbar spine as compared with fluoroscopically guided TFESI. Although the radiation exposure for CT-guided TFESI was ten times that of fluoroscopically guided TFESI, it was associated with a significantly lower cost per procedure.

The clinical outcomes found within our two groups are not in keeping with previous studies comparing the efficacy of fluoroscopically guided and CT-guided TFESI of the lumbar spine. Dietrich et al.’s analysis of 648 patients who underwent lumbar TFESI revealed no significant difference in patients’ reported outcomes at 1 day, 1 week or 1 month using the Patient Global Impression of Change scale, with nearly half of patients reporting clinically relevant improvements in their symptoms [3]. Similar clinical outcomes were found in a systematic review of the clinical efficacy of CT-guided TFESI, though the authors note the absence of randomised control trials and the presence of potential reporting biases in the available literature [6, 9–11]. A higher success rate of 92.9% was found by Sun et al., however, a significant proportion of this study’s population had experienced symptoms for less than three months, which, when combined with the lack of control group, may represent a confounding factor (the number of these patients who would have had spontaneous resolution of their symptoms is unclear) [12]. Due to this, at our institution, patients must have had symptoms present for at least 3 months that are resistant to conservative measures including analgesia and physiotherapy.

The differences between these studies and the one reported here may be due to the primary outcome measures used and the influence of confounding factors. Due to the unavailability of patient-reported outcomes measures in our dataset, we used the 6-week follow-up appointment outcome as a surrogate marker for clinical efficacy. As such, patients who had received clinically relevant levels of symptom relief may have been missed through this method if the surgeon decided that the patient should receive further follow-up or operative management. Furthermore, given the unblinded nature of the follow-up, the operating surgeons may have been resistant to discharge a patient upon whom they had performed an intervention. This is suggested by our finding that the only outcomes that demonstrated statistical significance were those of discharge and further follow-up.

In addition, our results revealed TFESI of the lumbar spine to be a safe procedure, with no documented complications in our patient cohort. Although spinal cord injuries have been reported in patients undergoing TFESI, these represent rare cases and are thought to have been the result of steroid injection into a radicular artery causing either embolization or direct neurotoxicity [13–16]. These cases highlight the necessity of a test dose of contrast medium to identify a potentially aberrant needle position, allowing the procedure to be aborted to prevent complications from arising.

Though adherence to ‘As Low As Reasonably Achievable’ principals is widely used in interventional radiology procedures, we found the effective dose of radiation to be ten times higher in patients undergoing CT-guided TFESI when compared to fluoroscopically guided TFESI. This difference is similar to that found by Maino et al. following their analysis of 100 TFESI and facet joint block procedures [4]. A significant but far smaller difference was also found by Dietrich et al. [3]. This smaller radiation dose is likely due to the use of a radiation reducing protocol in this study, a technique which has been suggested to reduce effective radiation dose by up to 88% whilst maintaining ‘satisfactory’ image quality [17–20]. Although previous studies have suggested this approach is safe and reproducible, larger prospective randomised studies are needed to ensure these low dose protocols do not jeopardise clinical efficacy or patient safety.

Our study found that fluoroscopically guided TFESI was four times the cost of CT-guided TFESI. Though the majority of the cost difference can be attributed to utilising a fully staffed operating theatre for the procedure, it represents the real-world cost of this treatment modality at our institution. The use of theatres also resulted in a higher cost of medical indemnity as indemnity was required for an increased number of staff members, including both an orthopaedic spinal consultant and a consultant anaesthetist, which is standard protocol for our institution. Furthermore, with increasing demand for operating theatre space in multiple specialities, the opportunity cost associated with utilising a fully staffed theatre must also be considered. However, even when theatre and staffing costs were removed, CT-guided TFESI remained significantly less expensive (£132.60 vs £237.40). This represents an estimated cost if the fluoroscopically guided TFESI were to be performed in the radiology department, with the same staff as the CT-guided TFESI.

Pharmacy costs were found to be significantly higher in the CT-guided TFESI group when compared to the fluoroscopically guided TFESI group. This is likely due to timeframe in which the long-acting corticosteroids were ordered prior to use—in theatres there are sufficient facilities availability to enable advanced ordering and planning, thus reducing the cost when compared to the radiology department where drugs had to be order on the morning of procedure at a higher cost.

Our cost calculations included estimations of life-span of each imaging machine to determine cost-per-use. As fluoroscopy imaging is used less frequently, it was deemed to be similar in imaging costs to CT, as the cost of CT imaging is offset by the greater frequency of use by other services throughout its life-span. The costs of CT-guided TFESI calculated in our study are higher than previous estimates. Mauer et al. estimated the cost of CT-guided TFESI at their institution in Germany to be €88, of which €23 was attributed to imaging use, €35 in staffing cost and €41 for disposables [21]. Differences between our study and these estimates can be attributed to differences in operational and equipment costs between the United Kingdom and Germany, and the of procedure time to estimate pro-rated costs. Though this study included idle time during the procedure in their calculations, their calculations likely underestimate the proportional time the CT scanner and staff must be ‘booked’ for to complete the procedure as part of a procedural list. In addition, this study did not include estimated cost associated with overheads, indemnity or use of wards and recovery which consisted of over one quarter of our total cost.

### Limitations

Although the results of this study provide valuable information regarding the efficacy, safety and cost of TFESI, the generalisability of these findings is hindered by several limitations. The retrospective nature of this study and the lack of randomisation limit the validity of conclusions that can be drawn regarding clinical outcomes. As this was a single-centre study with a limited number of proceduralists, the efficacy, safety and costs at other institutions may differ due to variable levels of experience and differences in operating costs. One important factor that contributes to clinical decision making for CT-guided procedures is the limited availability of the scanning facilities in many centres, due to prioritised imaging studies for emergencies. This may mean greater clinical utility for fluoroscopically guided TFESI when fluoroscopy facilities are more readily available. As stated above, multicentre prospective studies are required to address these issues and provide further evidence as to the clinical efficacy of these procedure modalities. Another approach increasingly adopted by other subspecialties is the establishment of national procedure databases, providing large volumes of multicentre multi-surgeon data including patient reported outcome measures and complication rates. Application of this approach to TFESI would provide high-quality data and allow effective comparison of these two treatment modalities.

Though the method used in this study to calculate the effective dose is widespread, it does represent an indirect technique. It is unclear how these doses differ from direct measurements, for example with a participant dosimeter, or between patients of different BMIs or genders. In addition, we did not assess the radiation dose received by the operator, something that has previously been suggested to be significantly higher for CT-guided procedures compared with other procedures.

## Conclusion

At our institution, CT-guided TFESI was associated with a higher 6-week discharge rate, a lower cost, but a ten times higher radiation dose as compared with fluoroscopically guided TFESI. Prospective randomised studies or national databases are required to effectively compare the clinical efficacy of these procedures and to investigate how the radiation dose associated with CT-guided TFESI can be reduced without jeopardising efficacy or safety.

## Data Availability

Data and material will be available on request to the corresponding author.
